# *SLC2A5* promotes lung adenocarcinoma cell growth and metastasis by enhancing fructose utilization

**DOI:** 10.1038/s41420-018-0038-5

**Published:** 2018-02-26

**Authors:** Yuanyuan Weng, Xueyu Fan, Yongfeng Bai, Siwei Wang, Hui Huang, Huimin Yang, Jin Zhu, Feng Zhang

**Affiliations:** 1 Department of Clinical Laboratory, Core Facility, Quzhou People’s Hospital, Quzhou, Zhejiang China; 2 Department of Pharmacology, Quzhou People’s Hospital, Quzhou, Zhejiang China; 3 Shanghai Biomedical Laboratory, Shanghai, China

## Abstract

The metabolism of cancer cells is highly plastic. Cancer cells can change their preference for nutrient uptake under nutrient stress. Fructose is one of the most common carbohydrates in diet and its metabolism is also involved in the development and progression of tumors. GLUT5, encoded by *SLC2A5*, is the specific fructose transporter in mammalian cells. In this study, we found that *SLC2A5* is significantly upregulated in lung adenocarcinoma (LUAD) patients and overexpression of *SLC2A5* is highly correlated with poor prognosis of LUAD patients. The expression of *SLC2A5* determined fructose uptake and utilization efficacy in LUAD cells. GLUT5 is critical for the survival of LUAD cells in fructose-containing culture medium. Depletion of *SLC2A5* undermined cell proliferation and invasion meanwhile increased cell apoptosis. Overexpression of *SLC2A5* enhances cell proliferation, migration, invasion, and tumorigenic. Compared to glucose, fructose is prone to strengthen intracellular-free fatty acid accumulation and ATP production. Moreover, inhibition of GLUT5 by specific small chemical inhibitor sensitizes LUAD cells to paclitaxel treatment. Taken together, our results suggest that GLUT5 could be a potential target alone or combination with other treatment for lung cancer therapy.

## Introduction

Alteration of cellular metabolism is one of the hallmarks of cancer cells^1, 2^. Mutations of oncogenes and tumor suppressor genes drive somatic cells to tumor cells, which also reprogram the intracellular metabolic pathways to provide building blocks and energy required for rapid cell proliferation or survival in harsh environment. For example, even in the case of sufficient oxygen supply, most cancer cells rely on aerobic glycolysis instead of mitochondrial oxidative phosphorylation to generate the energy needed for cellular processes, a phenomenon termed “Warburg effect”^3^. Although Warburg effect used to be considered as dominant metabolic feature for cancer cells, it has now become clear that the Warburg effect represents only a fraction of the metabolic rearrangements that accompany malignant transformation^4^. The metabolic adaptation of tumor cell is highly complex and plastic, not only genetic factors but also the nutrient availability in surrounded environment can promote cancer cells to adjust the activity of different metabolic pathways, utilizing alternative nutrients as sources of carbon and nitrogen for their biological functions^5, 6^.

Fructose is one of the most common carbohydrates in diet. In the past, humans consume a relatively small amount of fructose from fruits. However, the amount of fructose in people’s diet has increased significantly since the 1970s^7^. At present, fructose accounts for approximately 5–15% of daily calorie intake^8, 9^. Fructose is also widely used in elderly and children’s food. In recent years, due to the significant intake of fructose in the daily diet, its impact on many diseases, including cancers, has attracted attention of scientific researches. In addition, fructose metabolism is also involved in the development and progression of tumors^10^. Abnormal active glycolytic metabolism can lead to a serious shortage of glucose levels in the tumor microenvironment. In this case, how to adjust the metabolism of tumor cells and maintain sufficient carbon uptake to maintain cell proliferation is critical for tumor progression. Studies have shown that acute myeloid leukemia (AML) cells utilize fructose as a substitute to promote cell proliferation in the absence of glucose^11^. Not only that, the intake of fructose is associated with an increased risk of breast cancer, pancreatic cancer, and small bowel cancer^12^. Pancreatic cancer cells preferred fructose in its nucleic acid synthesis and fructose can promote pancreatic cancer proliferation. Increased fructose metabolism can promote pancreatic tumor growth by increasing the pentose phosphate pathway flux and protein synthesis^10^. Studies have suggested that fructose may increase the risk of breast cancer progression and metastasis by inducing the production of lipoxygenase-12 and a related fatty acid 12-HETE in breast cancer cells^13^. However, relative to glucose metabolism, our knowledge of fructose metabolism in tumor pathology and the underlying mechanism is very limited.

GLUT5 has very low affinity for other carbohydrates such as glucose and galactose, and is a specific fructose transporter^14, 15^. GLUT5 is encoded by the *SLC2A5* gene of the SLC2 family^16^. The expression of *SLC2A5* is elevated in breast cancer cell lines MCF7 and MDA-MB-231, and is associated with higher fructose uptake rate^17^. Recent studies have shown that the expression of GLUT5 in tumor cells of patients with AML increased and is negatively correlated to the prognosis of patients^11^. It is noteworthy that knockdown of GLUT5 in breast cancer cells and AML cells can significantly reduce fructose uptake and inhibit tumor cell proliferation^11, 12^. Our preliminary analysis showed that the expression of *SLC2A5* was upregulated in non-small-cell lung cancer (NSCLC) samples compared to normal lung tissue, but the implication of *SLC2A5* upregulation in lung cancer was largely unknown.

In this study, we showed that *SLC2A5* is significantly upregulated in lung adenocarcinoma patients and overexpression of *SLC2A5* is highly correlated with poor patient survival. The expression of *SLC2A5* determined fructose uptake in LUAD cells. Functionally, GLUT5 is critical for the survival of LUAD cells in fructose-containing culture medium. Depletion of *SLC2A5* impairs cell proliferation and migration, while overexpression of *SLC2A5* enhances cell phenotypes in these regards. In addition, inhibition of GLUT5 by specific small chemical inhibitor enhances the sensitivity of LUAD cells to paclitaxel treatment. Our results suggest that fructose uptake could be a potential target for LUAD detection. Blockage of GLUT5 alone or combination with other treatment would be meaningful for lung cancer therapy.

## Results

### *SLC2A5* is overexpressed in NSCLC and its overexpression associates with poor prognosis of lung adenocarcinoma

**Fig. 1:**
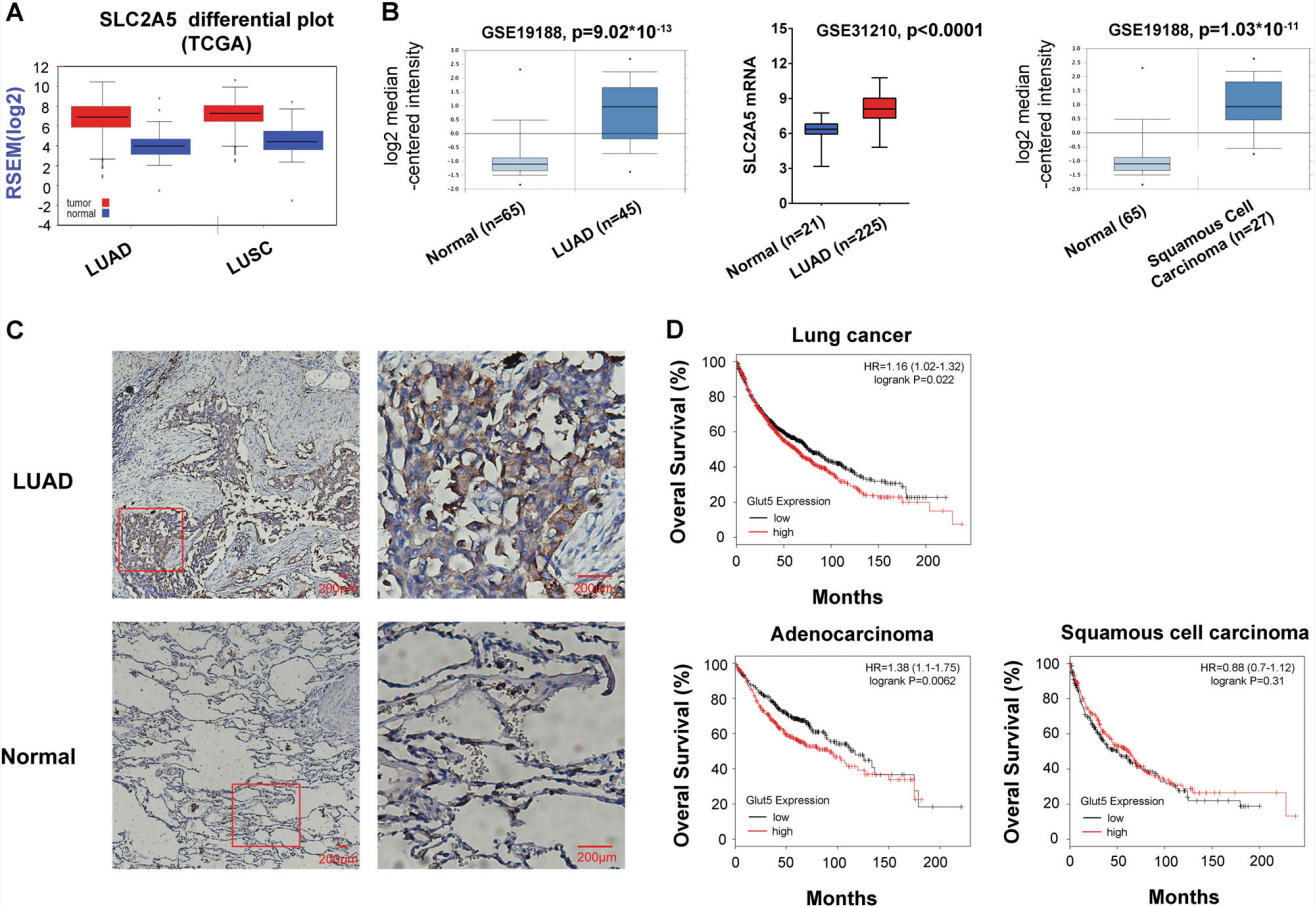
*SLC2A5* is overexpressed in NSCLC and is correlated with poor prognosis of lung adenocarcinoma. **a** *SLC2A5* RNA abundance in TCGA database. **b** *SLC2A5* mRNA expression in GEO datasets. **c** Immunochemistry staining of lung adenocarcinoma and normal lung tissue sections by Glut5 antibody. **d** Correlation of *SLC2A5* expression with patient survival in lung cancer. Probability of overall survival with lung cancer (upper), lung adenocarcinoma and squamous cell carcinoma (lower) were obtained from Kaplan–Meier Plotter/lung cancer (http://www.kmplot.com/lung). Statistical analysis was performed with the log-rank test

First of all, we determined *SLC2A5* expression level in normal and cancerous lung tissue by bioinformatics analyses. RNA-Seq data from TCGA revealed expression of *SLC2A5* was higher in both lung adenocarcinoma and squamous cell carcinoma (SCC) compared to normal lung tissue (Fig. 1a). Microarray data from Oncomine and extracted from GSE31210 also supported *SLC2A5* was significantly elevated in lung adenocarcinoma and SCC (Fig. 1b). Furthermore, we detected Glut5 expression in paraffin-embedded tissue sample using GLUT5-specific antibody. Indeed, GLUT5 protein was strongly expressed in lung cancer tissue, while the staining in normal tissue was much weaker (Fig. 1c). Of importance, analysis of correlation between *SLC2A5* expression and patient prognosis from microarray database revealed that *SLC2A5* overexpression was associated with poor prognosis in lung cancer a whole (*p* = 0.022) and in lung adenocarcinoma (*p* = 0.0062) patients, but such association did not exist in lung SCC (*p* = 0.31) (Fig. 1d).

### GLUT5-mediated fructose utilization in lung adenocarcinoma cells

**Fig. 2:**
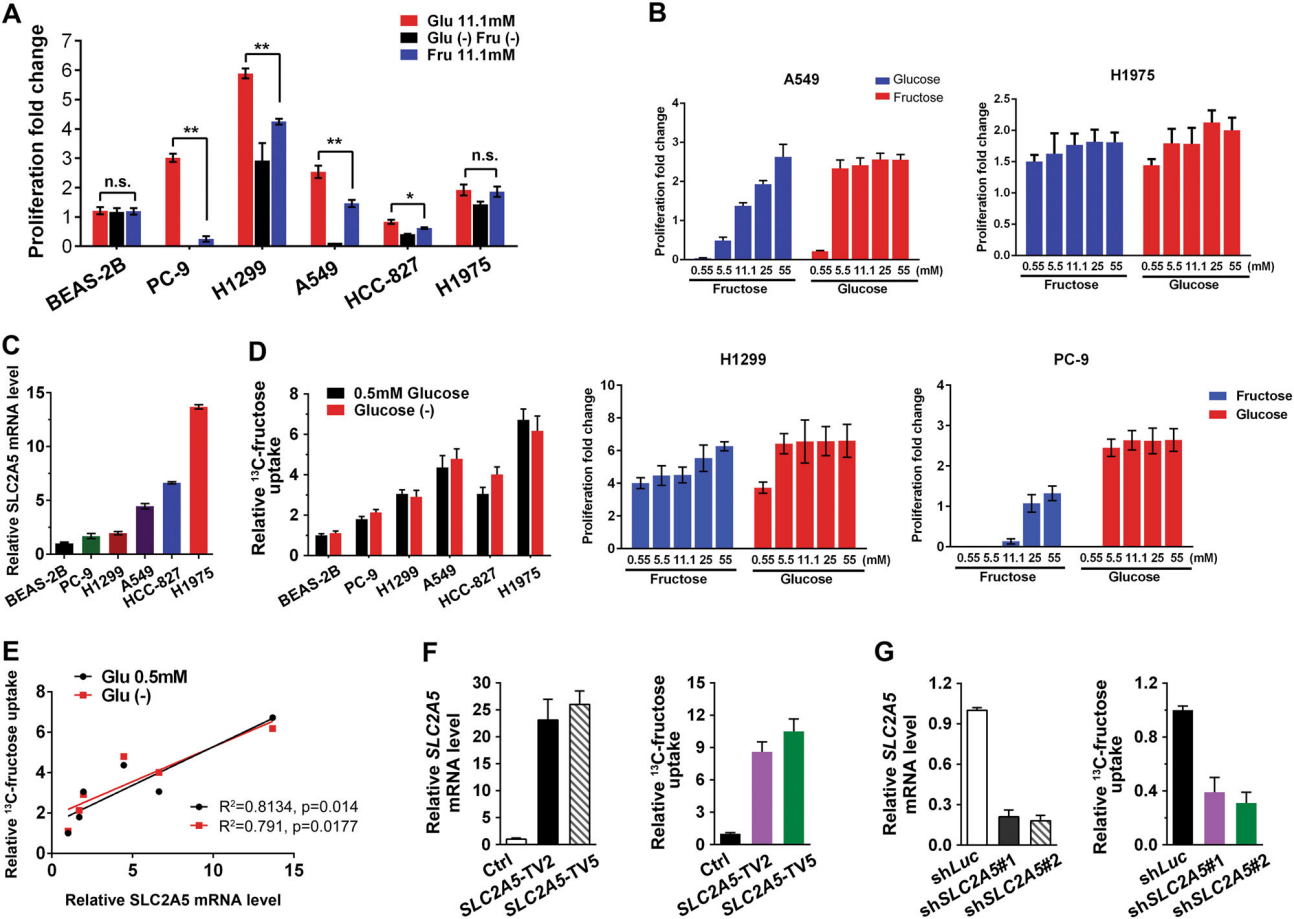
GLUT5-mediated fructose utilization by lung adenocarcinoma cells. **a** LUAD cell utilized fructose as alternative nutrient of glucose. Cells were grown in the media for 72 h. The proliferation of LUAD cells and normal lung cell line under the different nutrient conditions were measured. *p-*values were obtained by comparison with the cell proliferation in glucose media. **b** Fructose dependent proliferation of LUAD cells was determined by both individual cell property and fructose abundance. **c** GLUT5 expression in various LUAD cells and normal lung cell line. Relative GLUT5 expression was calculated through dividing the value of Glut5 by the value of actin signal intensity. **d** ^13^C-Fructose uptake by LUAD cells and normal lung cell line under low glucose or glucose-free culture conditions. **e** The correlation of *SLC2A5* expression and fructose uptake. Linear regression was evaluated by GraphPad Prism software. **f** Ectopic expression of *SLC2A5* enhanced ^13^C-fructose uptake in A549 cells. **g** Uptake of ^13^C-fructose was inhibited by *SLC2A5* knockdown in A549 cells

Notwithstanding, *SLC2A5* was overexpressed in lung adenocarcinoma and the expression was associated with prognosis, it is uncertain if lung adenocarcinoma cells utilize fructose as alternative nutrient. GLUT5, encoded by the *SLC2A5* gene, has been reported to be a highly selective transporter for fructose uptake in various cells. Recently, its function for fructose utilization in AML was studied by Chen and his colleagues. Herein, we tested the effect of fructose as major nutrient on LUAD cell growth. Different LUAD cell lines had diverse sensitivities to glucose deprivation, while in general fructose was an effective alternative nutrient of glucose. In three of the six cell lines we tested, 11.1 mM fructose was less effective to cell proliferation compared with 11.1 mM glucose (Fig. 2a). We hypothesized it was due to the different sensitivity of cells to those two kinds of sugars. To confirm our hypothesis we measured cell proliferation of LUAD cells under various glucose and fructose concentrations. The saturate concentration of glucose and fructose for cell lines differed from each other. As a whole, fructose influenced LUAD cell proliferation in a much wider range than glucose (Fig. 2b). To further demonstrate the relationship between *SLC2A5* expression and fructose utilization, we measured mRNA abundance of *SLC2A5* in various LUAD cell lines (Fig. 2c) and fructose uptake under conditions of limited glucose or glucose-free medium (Fig. 2d). The correlation of *SLC2A5* expression level and fructose uptake was assessed by linear regression. The result indicated fructose uptake was largely determined by the amount of GLUT5 (Fig. 2e). Furthermore, we overexpressed *SLC2A5* in A549 cells. Fructose uptake was obviously increased under higher *SLC2A5* expression condition (Fig. 2f). Conversely, fructose uptake was dramatically inhibited when GLUT5 was depleted by lentivirus-mediated shRNA (Fig. 2g).

### GLUT5 is required for the survival of LUAD cells in fructose-containing medium

**Fig. 3:**
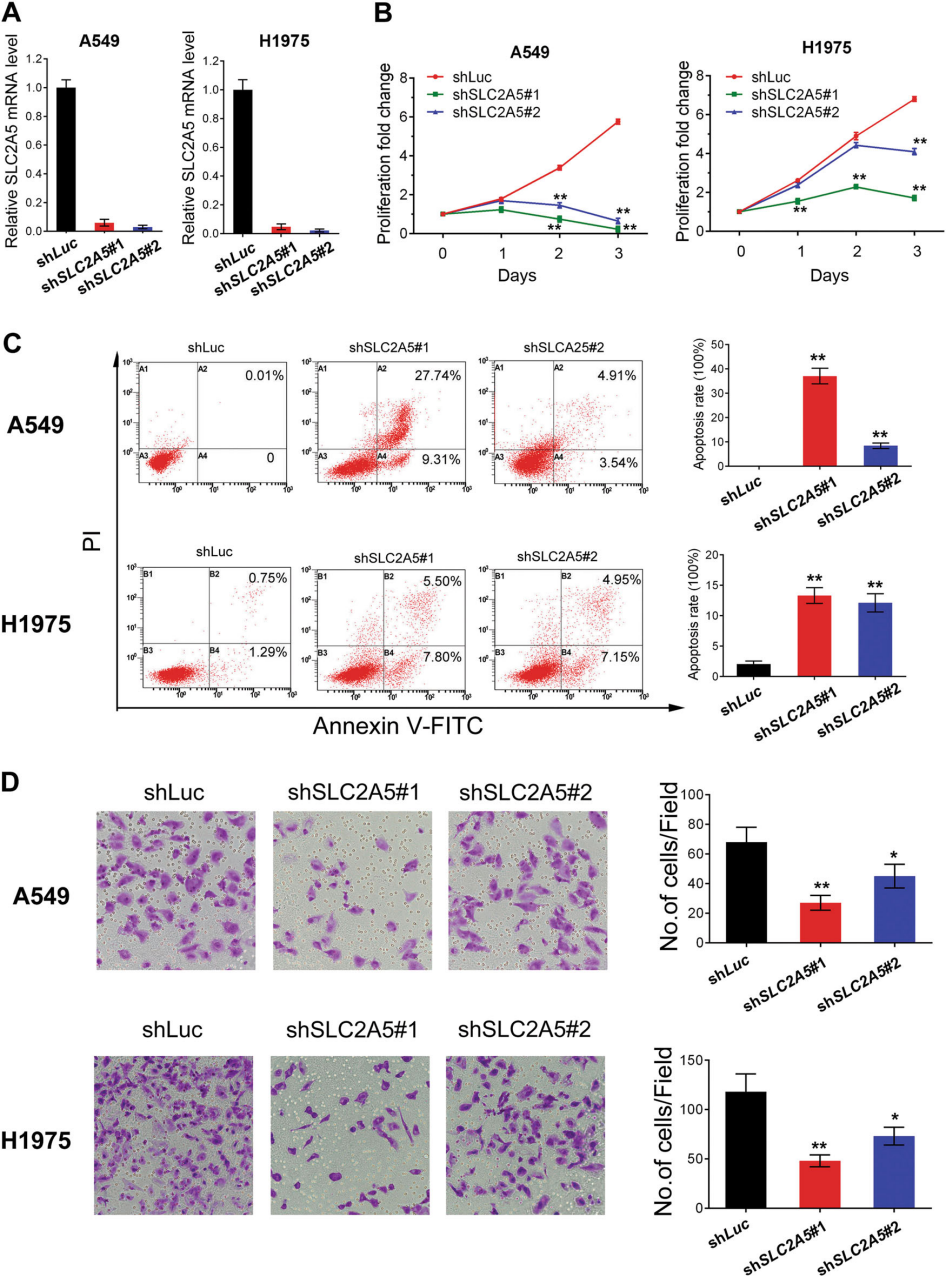
*SLC2A5* is crucial for LUAD cell survival and invasion in fructose medium. **a** Confirmation of *SLC2A5* knockdown in A549 and H1975 cells by shRNA. Cells were collected and lyzed 48 h after transdution with sh*Luc* or sh*SLC2A5*. **b** The proliferation of LUAD cells after *SLC2A5* depletion. Growth curves were generated based on counts of indicated cells. **c** Lack of sh*SLC2A5* triggered cell apoptosis in LUAD cells. Cells were stained by PI and FITC-Annexin-V. Apoptosis was measured by flow cytometry. Left, representative images were shown. Right, the summary of three independent experiments. **d** Effect of *SLC2A5* on cell invasion was evaluated in LUAD cells by Transwell invasion assay. sh*SLC2A5*-expressing cells showed obviously lower penetration rate through the Matrigel-coated membrane compared with control. *N* = 3; **p < 0.01

To study the role of GLUT5-mediated fructose utilization in cancer cell survival, we depleted *SLC2A5* expression by shRNAs. The effect of shRNA-mediated knockdown was examined by real-time PCR and western blot (Fig. 3a). Cells were cultured in media containing 25 mM fructose for 4 days and the proliferation index was counted. Lack of *SLC2A5* significantly affected cell proliferation in fructose-containing medium (Fig. 3b). Cells were stained by PI and FITC-Annexin-V and the apoptosis was detected by flow cytometry. *SLC2A5* depletion induced apoptosis prominently (Fig. 3c). Moreover, knockdown of *SLC2A5* reduced the ability of cell invasion revealed by transwell assay (Fig. 3d).

### Ectopic expression of *SLC2A5* exacerbated LUAD cell phenotype in medium containing fructose

**Fig. 4:**
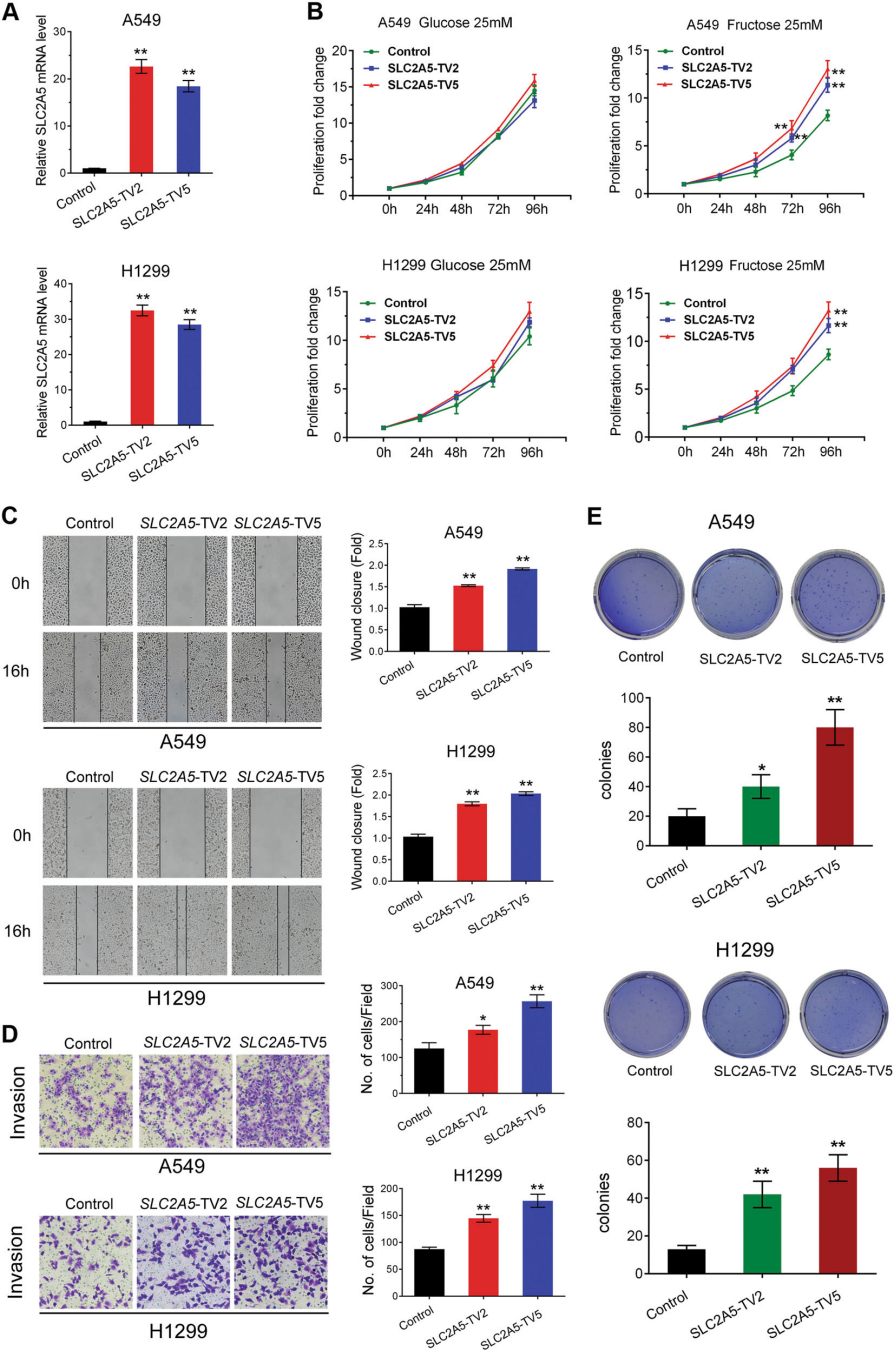
Ectopic expression of *SLC2A5* exacerbates the phenotypes of LUAD cells. **a** Measurement of SLC2A5 overexpression in A549 and H1299 cells transduced with lentiviruses of expressing EGFP (control) and two different *SLC2A5* transcript variants, transcript variant 2 (TV2) and 5 (TV5) by real-time PCR. **b** Proliferation of A549 and H1299 cells expressing control or *SLC2A5* transcript variants in medium containing 25 mM glucose or fructose. **c** Overexpression of *SLC2A5* increased mobility of LUAD cells. **d** Ectopic expression of *SLC2A5* enhanced cell invasion of LUAD cells in medium containing 25 mM fructose. **e** Colony formation of LUAD cells expressing control or *SLC2A5* transcript variants in soft agar fed with complete medium containing 25 mM fructose. *N* = 3; *p < 0.05; **p < 0.01

According to our previous data, *SLC2A5* overexpression promoted fructose uptake in LUAD cells, while the physiological effect was unclear. Therefore, we determined cell proliferation in *SLC2A5* overexpressed A549 and H1299 cells in medium containing glucose or fructose. Two transcripts of *SLC2A5* were cloned in lentiviral construct and stably expressed in A549 and H1299 cells (Fig. 4a). Ectopic expression of *SLC2A5* remarkably promoted cell proliferation in fructose-containing medium rather than medium containing glucose, indicating high specificity of *SLC2A5* for fructose uptake and utilization (Fig. 4b). Scratch assay showed that *SLC2A5* enhanced migration of A549 and H1299 cells (Fig. 4c). The invasion ability was also increased in *SLC2A5* overexpressed cells (Fig. 4d). Furthermore, the tumorigenic ability of A549 and H1299 cells was significantly enhanced by *SLC2A5* overexpression (Fig. 4e). Taken together, ectopic expression of *SLC2A5* exacerbated LUAD cell phenotype under fructose-containing culture condition.

### Metabolic differentiation between glucose and fructose

**Fig. 5:**
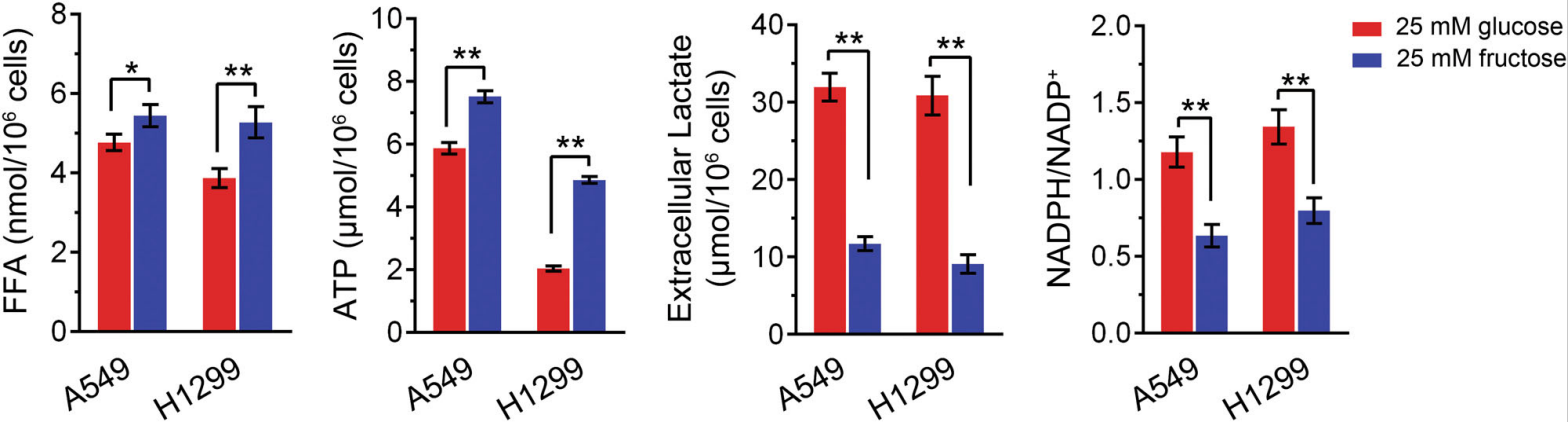
Metabolic differentiation between glucose and fructose. A549 and H1299 cells were cultured in medium containing 25 mM glucose or fructose. Cells were harvested and lyzed for FFA, ATP, and NADPH examination. Culture medium was collected for extracellular lactate determination. The data represented results from three experiments. **p < 0.01

Both glucose and fructose supported LUAD cell growth with different efficacy. To further demonstrate metabolic differentiation between glucose and fructose, we cultured LUAD cells in medium containing 25 mM glucose or fructose. The examination of FFA and NADPH revealed that fructose metabolism facilitated intracellular FFA accumulation while decreased NADPH consumption. Fructose induced more ATP and less lactate production compared to glucose in both A549 and H1299 cells (Fig. 5).

### Pharmacological blockage of fructose utilization potentiates efficacy of paclitaxel treatment

**Fig. 6:**
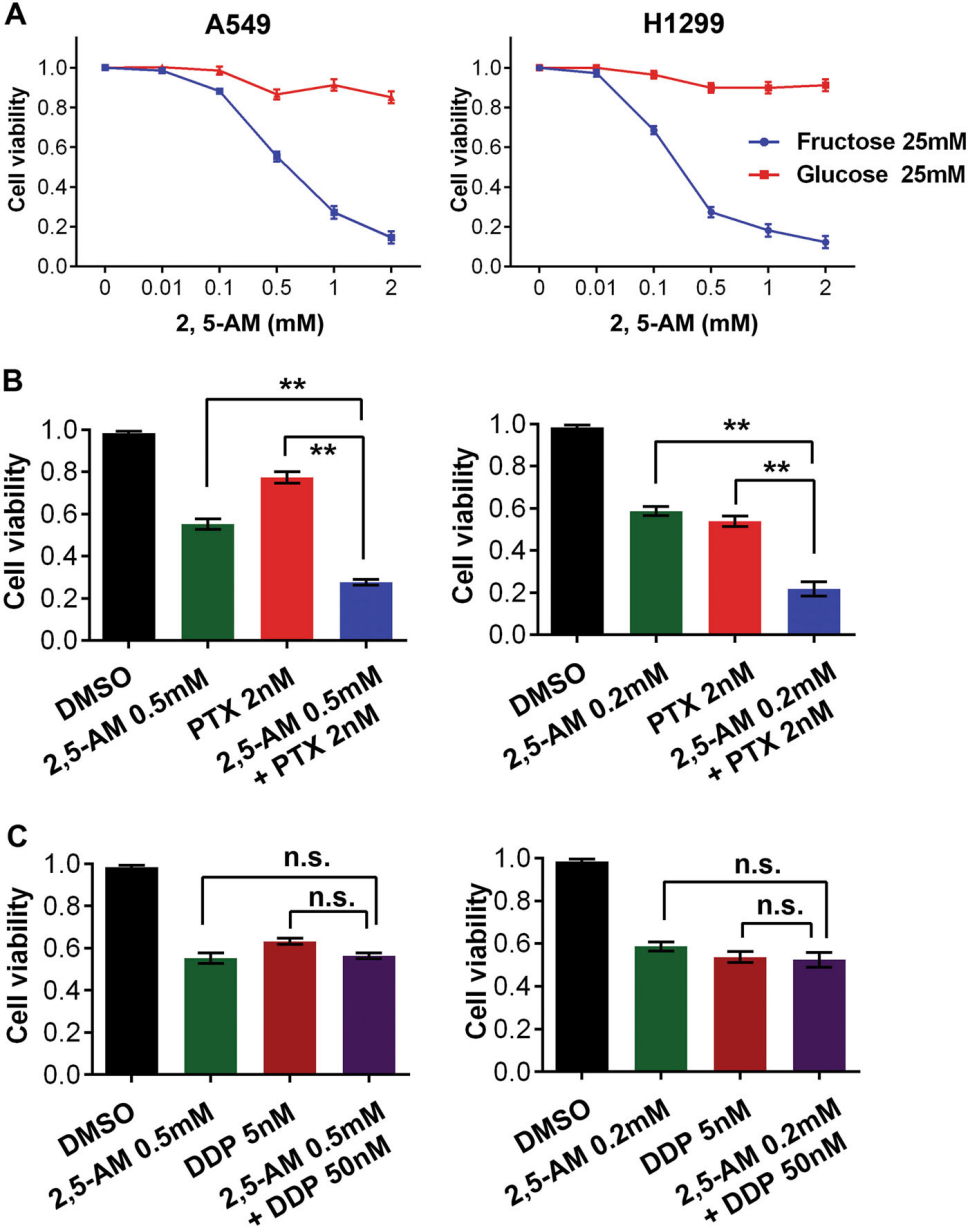
Pharmacological blockage of fructose utilization potentiates efficacy of paclitaxel treatment. **a** The effect of GLUT5 inhibitor, 2,5-AM, on A549 and H1299 cells cultured in medium containing 25 mM glucose or fructose. **b** The synergistic effect of 2,5-AM and paclitaxel in treatment of A549 (left) and H1299 (right) cells. **c** The effect of cisplatin treatment was not significantly enhanced by combining with 2,5-AM in A549 (left) and H1299 (right) cells

To determine the role of GLUT5 in current lung cancer therapy, we combined the inhibition of GLUT5 activity and chemotherapeutic treatment in LUAD cells. Inhibition of GLUT5 activity was achieved by 2,5-anhydro-D-mannitol (2,5-AM), a fructose analog with high affinity for GLUT5. GLUT5 inhibition significantly decreased cell viability of LUAD cells cultured in fructose-containing medium with little or no influence on cells under glucose culture condition (Fig. 6a). Next, we treated A549 and H1299 cells with a combination of commonly used chemotherapeutic drugs, paclitaxel and cisplatin. The efficacy was better in both cell lines when 2,5-AM was combined to paclitaxel, comparing to single drug treatment (Fig. 6b, Fig. S1). However, no synergistic effect was observed within the combination of 2,5-AM and cisplatin (Fig. 6c).

### Regulation of *SLC2A5* expression in LUAD

**Fig. 7:**
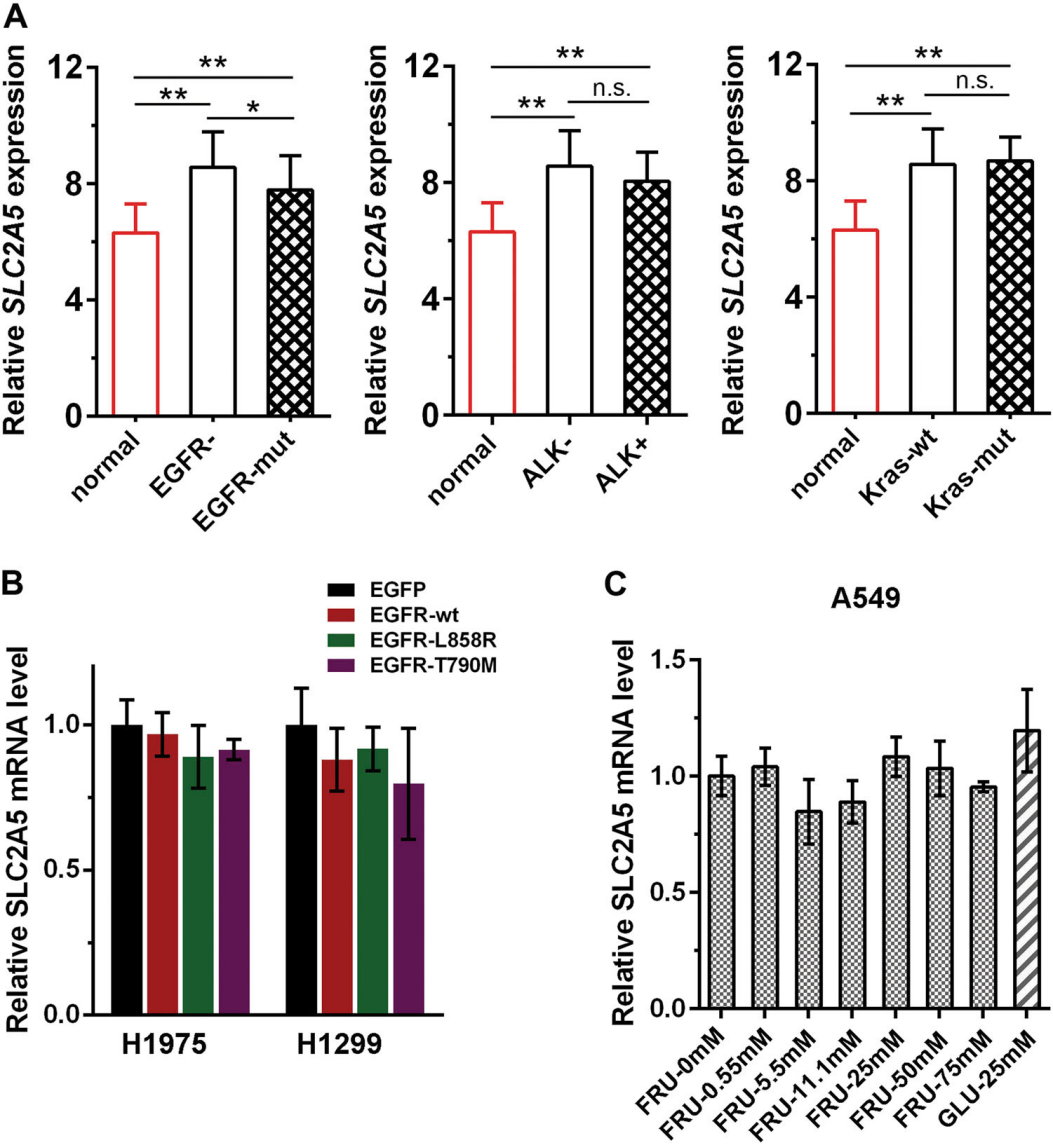
Regulation of *SLC2A5* expression in LUAD. **a** The samples from GSE31210 dataset were classified by *EGFR*, *ALK*, or *KRAS* status and the expression level of SLC2A5 was analyzed. *p < 0.05; **p < 0.01; n.s. no significance. **b** SLC2A5 expression was not influenced by EGFR activation. Wild-type or mutated active EGFR was introduced into LUAD cells. The expression of SLC2A5 was examined by real-time PCR (*N* = 3). **c** SLC2A5 expression was not induced by fructose or glucose in A549 cells. Cells were cultured in media containing indicated concentration of fructose or glucose for 48 h. The expression of SLC2A5 was examined by real-time PCR (*N* = 3). FRU fructose, GLU glucose

The expression of *SLC2A5* is reported to be regulated by many factors such as substrate level, tumor hypoxia, oncogene, inflammatory factor TNF-α, and hormone levels (insulin, thyroxine, etc.) in other tumors, but its modulation in lung cancer remain unknown. We tried to explore whether the status of oncogene was associated with *SLC2A5* expression. The samples from GSE31210 dataset were classified by *EGFR*, *ALK*, or *KRAS* status and the expression level of *SLC2A5* was analyzed. *SLC2A5* expression was significantly higher in tumor samples compared to normal tissues despite the status of *EGFR*, *ALK*, or *KRAS*. However, only *EGFR* status seemed to affect the abundance of *SLC2A5*, which was reduced in *EGFR*-mutated tumor samples (Fig. 7a). To confirm the relation between *EGFR* activation and *SLC2A5* expression, we mimicked the situation by ectopic expression of EGFR in LUAD cells. No significant change of *SLC2A5* expression was observed when different activities of EGFR were introduced into H1975 and H1299 cells (Fig. 7b). We also examined if fructose, the substrate of GLUT5, could influence the expression of *SLC2A5*. Likewise, we could not see any significant effect of fructose or glucose on *SLC2A5* expression in A549 cells (Fig. 7c).

## Discussion

Lung cancer results in the world’s highest incidence of morbidity and mortality in cancer. More than 85% of those cases are currently classified as NSCLC. The predicted 5-year survival rate of NSCLC is only 15.9%^18^. Nonetheless, the figure has only marginally improved during the past few decades. NSCLC is currently defined by pathological characteristics. The two predominant NSCLC histological phenotypes are adenocarcinoma (LUAD; ~50%) and SCC (~40%). In general, LUAD arise in more distal airways, whereas SCC arise in more proximal airways and are more strongly associated with smoking and chronic inflammation than LUAD^18, 19^. In our study, *SLC2A5* is overexpressed in both LUAD and SCC, but the effect seems not identical. In terms of overall survival, only adenocarcinoma is significantly associated with the expression levels of *SLC2A5*, implying that metabolism of cancer cell is complicated and its dependency on a specific nutrient is likely influenced by cell origin and oncogenic drivers. Not only different cell types of NSCLC cells, the sensitivity on fructose varies a lot even among LUAD cells. On the one hand, the same fructose concentration has different effects on accelerating cell growth (Fig. 2a). On the other hand, the saturated fructose concentration, which maximizes its growth effect, varies widely among cells (Fig. 2b). This might be a reflection of variation of GLUT5 abundance in different LUAD cell types, which largely determined the efficacy of uptake and utilization of fructose.

Unlike glucose, LUAD cells tend to utilize fructose for the synthesis of FFAs and ATP (Fig. 5). The observation that intracellular NADPH in fructose containing medium is lower than in glucose medium is likely due to the consumption of NADPH for enhanced fatty acid synthesis. Similarly, the reduction of fructose-induced lactate production by LUAD cells is probably due to more ATP produced by mitochondrial oxidation after the cells take up fructose, resulting in a decrease in pyruvate that could be converted to lactate.

Cisplatin and paclitaxel are two kinds of the most important drugs for chemotherapy of lung adenocarcinoma^20^. The action mechanism of cisplatin and paclitaxel are totally distinct. Cisplatin interferes with DNA replication to kill fast proliferating cells^21^. Paclitaxel stabilizes the microtubule polymer and protects it from disassembly, which impairs mitotic spindle assembly, chromosome segregation, and cell division^22^. The metabolic effect of fructose on the physiology of cancer cells is comprehensive. Therefore, inhibition of fructose utilization might affect a bunch of crucial physiological nodes of cells, which finally determine the cellular phenotypes. The difference in synergistic effect of GLUT5 inhibitor between combination with cisplatin and paclitaxel could reflect the extent of overlapping in their intracellular action sites with GLUT5 inhibitor. However, the intrinsic mechanism still needs further study and may lead to novel drug regimen for lung cancer treatment in future.

So far, there have been several studies reported that the expression of *SLC2A5* is regulated by glucocorticoid, insulin, and its substrate fructose. Glucocorticoid hormone dexamethasone was suggested to induce nuclear translocation of glucocorticoid receptor and methylation of histone H3 at K4 and acetylation of histones H3 and H4 to promote *Slc2A5* gene expression^23, 24^. Insulin is capable of increasing the abundance of GLUT5 in L6 skeletal muscle cells via activation of the *Slc2A5* promoter^25^. In addition, fructose could modulate GLUT5 mRNA stability in differentiated Caco-2 cells by the cAMP pathway and Paip2 (PABP-interacting protein 2) binding^26^. Introducing fructose into the lumen increased nuclear RNA, mRNA, protein, and activity levels of Glut5 in adult wild-type mice consuming chow^27^. Nonetheless, the regulation of *SLC2A5* in lung cancer is still unknown. Our results showed that neither the commonest tumor-driver genes nor fructose significantly influenced *SLC2A5* expression in lung adenocarcinoma. Identification of factors controlling the expression of GLUT5 expression and activity in lung cancer is an open question and worth further study.

## Materials and methods

### Reagents

Fructose analog 2,5-Anhydro-D-mannitol (2,5-AM, #41107-82-8) was purchased from Cayman (St. Louis, MO, USA). Cisplatin was from Sigma-Aldrich (#SP600125, St. Louis, MO, USA). Paclitaxel was ordered from Sangon Biotech Co., Ltd. (#A601183-0100, Shanghai, China). GLUT5 antibody was generated by Yingji Bio (Shanghai, China).

### Patients and specimens

The tumor samples were from patients who did not receive any preoperative cancer treatment. The samples were collected from these patients after obtaining informed consent according to an established protocol approved by the Ethics Committee of Quzhou People’s Hospital.

### Cell lines

HEK293T lentiviral packaging cell was from Dr. Xiaolin Wu’s lab. Human bronchial epithelial cell BEAS-2B and all NSCLC cell lines PC-9, H1299, A549, HCC-827, H1975 were obtained from American Type Culture Collection (Manassas, VA) and were cultured in Dulbecco’s Modified Eagle Medium (DMEM, Thermo Fisher Scientific) supplemented with 10% fetal bovine serum (FBS, Gibco, Thermo Fisher Scientific). All cell lines were grown at 37 °C in a humidified incubator with 5% CO_2_.

### Plasmids

The control firefly luciferase shRNA (sh*Luc*) was a kind gift from Dr. Guangwei Du. The oligos of *SLC2A5*-specific shRNAs (sh*SLC2A5*#1 and sh*SLC2A5*#2) were synthesized in Genewiz (Suzhou, China) and cloned in pLKO.1 lentiviral vector. The target sequences for sh*Luc*, sh*SLC2A5*#1, and sh*SLC2A5*#2 are listed in Table 1. For ectopic expression of *SLC2A5* in LUAD cells, two *SLC2A5* variant transcripts were cloned and constructed into pCDH-CMV-MCS-EF1-puro lentiviral vector. The primers for *SLC2A5* cloning are listed in Table 1. Wild-type EGFR was cloned into pCDH-CMV-MCS-EF1-puro lentiviral vector by primers listed in Table 1. The plasmids for mutated EGFR expression were ordered from Addgene (#11012 and #32070) and subcloned to pCDH-CMV-MCS-EF1-puro vector.

**Table 1:** Oligonucleotides used in our current study

Oligonucleotides	Sequences (5′ to 3′)
*SLC2A5*-forward	ACGTTGCTGTGGTCTGTAACC
*SLC2A5*-reverse	CATTAAGATCGCAGGCACGATA
β-actin-forward	AGAGCTACGAGCTGCCTGAC
β-actin-reverse	AGCACTGTGTTGGCGTACAG
sh*Luc*	GATTTCGAGTCGTCTTAAT
sh*SLC2A5*#1	GCACTGCTCATGCAACAATTT
sh*SLC2A5*#2	CGCCACATCATTTGAGCTTAT
*SLC2A5*-TV2-forward	ATGGAGCAACAGGATCAGAGC
*SLC2A5*-TV2-reverse	CTATGTTGGCTCGGGACAGGA
*SLC2A5*-TV5-forward	ATGTACTTAGGGGAGCTG
*SLC2A5*-TV5- reverse	CTGTTCCGAAGTGACAG
*EGFR*-WT-forward	ATGCGACCCTCCGGGAC
*EGFR*-WT-reverse	TCATGCTCCAATAAAT

### Cell proliferation and viability analysis

Cells were seeded in five replicates of wells in a 96-well plate, and the relative cell numbers were counted every day by CCK-8 (#40203ES60, Qcbio Science & Technologies Co., Ltd., Shanghai, China) over 3 days. For viability measurement, cells were trypsinized, stained with trypan blue solution. Trypan blue-negative viable cells and positive dead cells were counted under a light microscopy.

### Cell migration and cell invasion assays

For cell migration assays, cells were plated into 6-well plates and cultured at 37 °C and 5% CO_2_ for 1 or 2 days to permit cell adhesion and the formation of a confluent monolayer. Then a 200 μL tip was used to introduce scratches with equal widths. The cell surface was then washed with serum-free culture medium for three times to remove dislodged cells. Wound closure at 0, 12, and 24 h after scratching were recorded. We chose three random sites to calculate the mean and standard deviation. Images were collected at 0 and 24 h. For cell invasion assays, transwells (24-well) coated with Matrigel (8-μm pore size; BD Biosciences, San Jose, CA, USA) were used. Cells were serum-starved overnight. After pre-incubating the transwell insert for 1 h with serum-free media at room temperature, a total of 2 × 10^4^ cells were then resuspended in 100 μL DMEM containing 1% FBS and added to the upper chamber, while 600 μL DMEM with 10% FBS was added to the lower chamber as a chemoattractant. Cell migration was determined from triplicates for each treatment according to the manufacturer’s protocol.

### Colony formation assay

Cells were plated in 6-well plates covered by soft agar at a density of 2500 cells per well and cultured for 14 days to allow colony formation, the medium was replaced with fresh medium every 3 days. After 2 weeks, the medium was removed and cell colonies were stained with crystal violet (0.1% in 20% methanol) for 15 min. Colonies were photographed, and the number of colonies was counted using ImageJ (National Institutes of Health, Bethesda, MD, USA) from three independent experiments.

### Real-time PCR

Total RNA was extraction from cells by Trizol Reagent (#DP424, Tiangen Biotech Co. Ltd., Beijing, China) according to the manufacturer’s protocol, and reverse-transcribed using Maxima Reverse Transcriptases (#EP0751, Thermo Fisher Scientific). Real-time PCR was performed in triplicate using SGExcel FastSYBR Mixture (#B532955-0005, Sangon Biotech Co., Ltd., Shanghai, China) on Roche LightCycler^R^ 480 Quantitative PCR System (Indianapolis, IN, USA). Primers are listed in Table 1. Relative expression levels was normalized to β-actin and calculated by 2-ΔΔct method. Each experiment was performed independently at least three times.

### Flow cytometry analysis of apoptosis

Cells were transduced with lentiviruses carrying control sh*Luc*, sh*SLC2A5*#1, or sh*SLC2A5*#2 twice and harvested by trypsin digestion 2 days after infection with lentiviruses. Cells were washed with ice-cold phosphate-buffered saline and then re-suspended in 400 μL Annexin V binding buffer. 5 μL Annexin V-FITC (#40302ES50, Qcbio Science & Technologies Co., Ltd., Shanghai, China) and 10 μL propidium iodide (PI) were added and incubated for 15 min. Apoptotic cells were analyzed by BD flow cytometer (BD Biosciences, San Jose, CA). For each sample, at least 10,000 cells were collected. Data was analyzed by FlowJo software (FLOWJO, Ashland, OR). Cells without staining or stained with Annexin V-FITC or PI only were used as compensation controls.

### Lentivirus production and transduction

The modification of targeted gene expression was mediated by lentivirus. Viruses were generated in 293T cells. To produce viruses, plasmids including the lentiviral vector containing shRNAs or cDNA fragements, pCMVR8.74, and pMD2.G were co-transfected into 293T cells using Lipofectamine Plus reagent (Thermo Fisher Scientific) according to manufacturer’s instruction. At 24 and 48 h post-transfection, virus-containing supernatants were collected and centrifuged at 3000 × *g* for 5 min to remove suspended target cells. The supernatants were mixed with polybrene at final working concentration of 10 μg/mL for infection. Medium containing virus was replaced with fresh growth medium 6 h after transduction. Cells were then selected with 2 μg/mL puromycin for 2 days. The expression of targeted genes was examined by real-time PCR.

### Free fatty acid (FFA) quantification

Aliquots of 106 cell equivalents were extracted by homogenization with 200 μL of chloroform-Triton X-100 solution (1% Triton X-100 in chloroform) in a microhomogenizer and centrifuged at 16,000 × *g* for 10 min. Liquid of organic phase (lower phase) was collect and air dried at 50 °C to remove chloroform. Then the remainder was further dried by vacuum dryer for 30 min to extirpate chloroform. The concentration of FFA was measured by a commercial kit (#K612, BioVision), using the colormetric assay (OD 570 nm) and standardized to 106 cells. The data from three independent experiments were included.

### NADPH determination

The NADPH/NADP ratio was determination using the NADP/NADPH assay kit (#K347, BioVision) according to the manufacturer’s instructions. Briefly, 4 × 10^6^ cells were extracted in 800 µL of the NADP/NADPH extraction buffer. Samples were aliquoted. 50 µL sample was transferred into 96-well plate and heated at 60 °C for 30 min to compose NADP. Then 100 μL of the NADP Cycling Mix (buffer + enzyme) was added. For total NADP/NADPH detection, the mixture was incubated at room temperature for 5 min to convert NADP to NADPH. Afterward, 10 µL NADPH developer was mixed and incubated in the dark for 2 h. Then the plate was read at OD450 nm, and the data was normalized to nmol/10^6^ cells using a standard curve.

### Lactate measurement

2 × 10^5^ cells were cultured in 6-well plate in medium containing 25 mM glucose or fructose for 48 h. The culture media was collected and centrifuged at maximum speed to remove any cell debris. Lactate content in the supernatant was analyzed in the department of clinical laboratory in our hospital.

### ATP measurement

ATP level was measured by ATP Determination Kit (Molecular Probes) according to the manufacturer’s instruction. Briefly, A549 and H1299 cells were cultured in 6-well plates under glucose or fructose-containing medium for 24 h. Cells were harvested and lysed by adding 150 µL of lysis buffer NET (20 mM Tris, 100 mM NaCl, 1 mM EDTA) with 0.5% Triton-X 100 per 10^6^ cells and kept on ice for 10 min. Cell lysates were centrifuged at 16,000 × *g* for 10 min. Supernatants were collected. The standard reaction solution was prepared according to manufacturer’s instruction. 5 µL of cell lysate for each sample was mixed with 100 µL ATP standard solutions. The ATP level was determined by measuring firefly luciferase luminescence and normalized to the number of 10^6^ cells.

### Measurement of ^13^C-labeled fructose uptake

To assess the fructose uptake, cells were cultured in glucose-free DMEM or DMEM containing 0.5 mM glucose supplemented with 10% FBS and 15 mM ^13^C-fructose (Cambridge Isotope Laboratories). After incubation for 72 h, cells were collected and ^13^C-fructose uptake was analyzed by GC-MS assays performed in Shanghai Biomedical Laboratory.

### Bioinformatic analysis

The expression of SLC2A5 in lung cancer was analyzed by the Broad Firehose database (http://firebrowse.org/) and Oncomine (www.oncomine.org). We evaluated the correlation of SLC2A5 expression levels to patient survival in lung cancer using the Kmplotter (kmplot.com/analysis). Patients were split by median SLC2A5 expression, and analysis was performed the lung dataset irrespective of grade, stage, or prior treatment regimen.

### Statistical analyses

SPSS 21.0 (SPSS, Inc., Chicago, IL, USA) was used for all statistical analyses. Significant differences between groups were determined using the Student’s *t-*test. Values are presented as the mean ± standard deviation. Differences were considered to be statistically significant at *p* < 0.05 and highly significant at *p* < 0.01.

## Electronic supplementary material

Figure S1

Supplementary Figure Legend
