# Supplementary figures and images for: SLC2A5 promotes lung adenocarcinoma cell growth and metastasis by enhancing fructose utilization
Source: Cell Death Discov. 2018 Feb 26;4:38. doi: 10.1038/s41420-018-0038-5 (PMC5841403; doi:10.1038/s41420-018-0038-5)

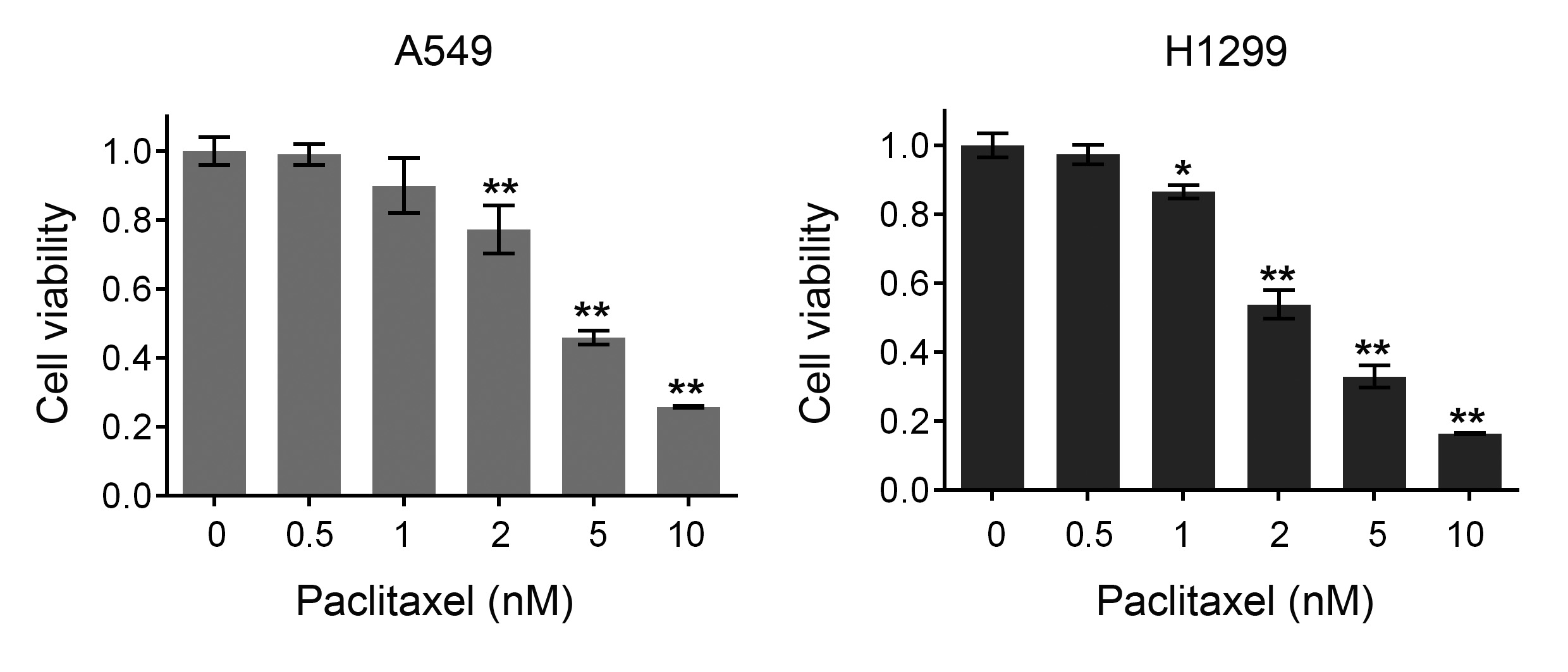

Supplement: Supplementary file 1 — Figure S1 [file 41420_2018_38_MOESM1_ESM.jpg]
