# Supplementary material for: SLC2A5 promotes lung adenocarcinoma cell growth and metastasis by enhancing fructose utilization
Source: Cell Death Discov. 2018 Feb 26;4:38. doi: 10.1038/s41420-018-0038-5 (PMC5841403; doi:10.1038/s41420-018-0038-5)
Supplement: Supplementary file 2 — Supplementary Figure Legend [file 41420_2018_38_MOESM2_ESM.docx]

**Fig S1. related to Fig 6. The dose effect of paclitaxel on the viability of A549 and H1299.** Cells were treated by indicated concentration of paclitaxel for 48 hr. The viability was measured by trypan blue exclusion assay. The concentration of 2 nM paclitaxel was chosen for further experiments.
